# Coexistence of homologous-type carcinosarcoma of the cervix with undifferentiated carcinoma of the endometrium: A case report with Immunohistochemical analysis and literature review

**DOI:** 10.1016/j.gore.2022.100924

**Published:** 2022-01-07

**Authors:** Giovanna Giordano, Elena Feiroli, Anna Maria Rodolfi, Serena Madaro, Roberto Berretta

**Affiliations:** a Department of Medicine and Surgery, Pathology Unit, University of Parma, Italy; b Department of Pathology, Guglielmo da Saliceto Hospital, Piacenza, Italy; c Department of Oncoly, Guglielmo da Saliceto Hospital, Piacenza, Italy; d Department of Medicine and Surgery, Unit of Surgical Sciences, Obstetrics and Gynecology, University of Parma, Italy

**Keywords:** Undifferentiated carcinoma of the endometrium, Dedifferentiated endometrioid adenocarcinoma, Malignant mixed Müllerian tumour, Carcinosarcoma

## Abstract

We report a case of undifferentiated carcinoma of the endometrium associated with malignant mixed Müllerian tumour of the uterine cervix. Immunohistochemical analysis with multiple markers was performed to demonstrate the coexistence of highly two aggressive components in the same uterus. Clinical data were collected and followed up, and a careful literature review was performed to establish the occurence of these components in a uterine malignancy.

## Highlights

• Cervical Carcinosarcoma is very rare and aggressive neoplasm.
• A cervical carcinosarcoma was diagnosed in a 60-year-old patient on cervical biopsies.
• Post-surgical study revealed a concomitant endometrial undifferentiated carcinoma.
• The adjuvant chemotherapy and radiotherapy were used to avoid a disease recurrence.
• The patient died after two months from post-actinic enteritis with intestinal occlusion.

## 1 Introduction

Undifferentiated carcinoma of the endometrium (UCAe) is a subtype of endometrial carcinoma and is included in the WHO classification of endometrial carcinomas (Tavassoli and Devilee, 2003). UCAe accounts for approximately 9% of endometrial carcinoma cases (Silva et al., 2007) and represents an extremely aggressive variant of endometrial carcinoma; it may occur in a pure form or in combination with a low-grade endometrioid adenocarcinoma (grade 1 or 2; e.g., dedifferentiated endometrioid adenocarcinoma) (Silva et al., 2006, Silva et al., 2007). In this occurrence, UCAe is often misdiagnosed as a grade 3 endometrioid adenocarcinoma or even a grade 2 endometrioid adenocarcinoma.

The recognition of the undifferentiated component in endometrial carcinoma is extremely important because its prognosis seems to be worse than that of grade 3 endometrioid adenocarcinoma (Silva et al., 2006). Herein, we report a case of UCAe associated with malignant mixed Müllerian tumour (MMMT) of the uterine cervix, which is a very rare malignancy (Kim et al., 2015, Iida et al., 2005). MMMT, which is also known as carcinosarcoma (CS), is a mixed tumour with an epithelial histologic component (carcinomatous) and a mesenchymal component that appears often in the corpus of the uterus, less frequently in the ovaries, and exceptionally in the cervix (Kim et al., 2015, Iida et al., 2005, Comert et al., 2017). Immunohistochemical analysis with multiple markers was performed both for cervical CS and UCAe to demonstrate the coexistence of two independent malignancies in the same uterus. In addition, clinical data were collected and followed up, and a careful literature review was performed to establish the frequency and prognostic significance of the combination of these rare malignancies.

## 2 Case report

A 60-year-old gravida 2, para 2 female was admitted to the Gynecologic and Obstetrics Unit of Parma Medical University (Italy) with a diagnosis of uterine cervix CS. Six months prior to this event, she was hospitalised at the Gynecologic and Obstetrics Unit of Piacenza Hospital (Italy) because of abnormal uterine bleeding.

The patient did not report any relevant medical history. Her last cervical smear was performed five years ago and was normal. A genital examination revealed atrophic external genitalia and a large vaginal polyploid lesion protruding from its cavity, which hindered the identification of the cervix and impeded speculoscopy.

Histological examination was performed on small fragments of the cervical lesion. A histopathological assessment of the material collected at the Pathology Unit of Piacenza Hospital (Italy) revealed a uterine cervix CS that is characterised by the presence of glandular epithelial and malignant stromal components. Pelvic magnetic resonance imaging (MRI) and contrast-enhanced computed tomography (CT) were performed to establish the developmental stage of the cervical neoplasm. MRI confirmed the presence of a cervical lesion that replaced entirely the endocervical wall entirely and involving both parameters, but was more evident at the right side. Posteriorly, the neoplasm was in direct contact with the rectum, and it was not possible to identify a cleavage plan. The lesion extended towards the body of the uterus, thus causing a stagnation of material in the uterine cavity. These findings were also associated with the presence of enlarged bilateral hypogastric lymph nodes.

Contrast-enhanced computed tomography (CT) of the thorax, abdomen, and pelvis confirmed the presence of a large uterine cervical neoplasm and enlarged bilateral hypogastric lymph nodes. There was no evidence of a mass or metastatic disease in the chest or abdomen. Therefore, the final stage of cervical neoplasm was IIIB (FIGO).

The patient was referred to the Oncology Unit of Piacenza Hospital (Italy), where she was treated with four cycles of neoadjuvant chemotherapy (carboplatin and Taxol). After neoadjuvant chemotherapeutic treatment, a CT and MRI study of the cervical neoplasm showed volumetric reduction measuring 4.6 cm × 3.4 cm vs. 5.9 cm × 4.2 cm. Furthermore, the hypogastric lymph nodes were slightly reduced in size.

Thereafter, the patient underwent radical hysterectomy with bilateral salpingo-oophorectomy, pelvic and *para*-aortic lymph-node dissection, and omentectomy at the Gynecologic and Obstetrics Unit of Parma Medical University (Italy) to establish a post-surgical staging of cervical neoplasm.

**Fig. 1:**
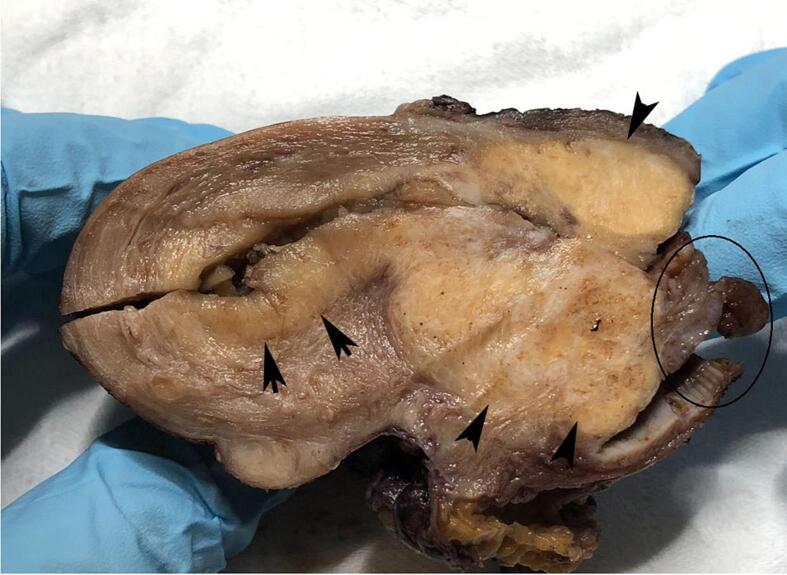
A macroscopic examination of the uterus showed a cervical polypoid lesion with superficial haemorrhage protruding from the uterine external orifice with a myxoid (circle) that was in continuity with an endocervical heterogeneous lesion with whitish and yellow ochre areas of the endocervical wall (arrow heads). The endometrium was replaced by a whitish lesion that seemed to superficially infiltrate the myometrium and extended up to the uterine isthmus (arrows). (For interpretation of the references to colour in this figure legend, the reader is referred to the web version of this article.)

Macroscopic examination showed that the uterus was slightly enlarged, and a cervical polypoid lesion with superficial haemorrhage measuring 4.5 cm × 3.0 cm in size was found protruding from the uterine external orifice. On the cut surface, this neoplasm had a myxoid appearance and was a heterogeneous lesion in continuity with whitish and yellow ochre areas that almost completely replaced the exocervical and endocervical walls. The endometrium was thickened and completely replaced by a whitish lesion that seemed to infiltrate superficially the myometrium and extended up to the uterine isthmus (Fig. 1).

The short segment of vaginal wall attached to the cervix, its mucosa, and the bilateral adnexa were unremarkable.

**Fig. 2:**
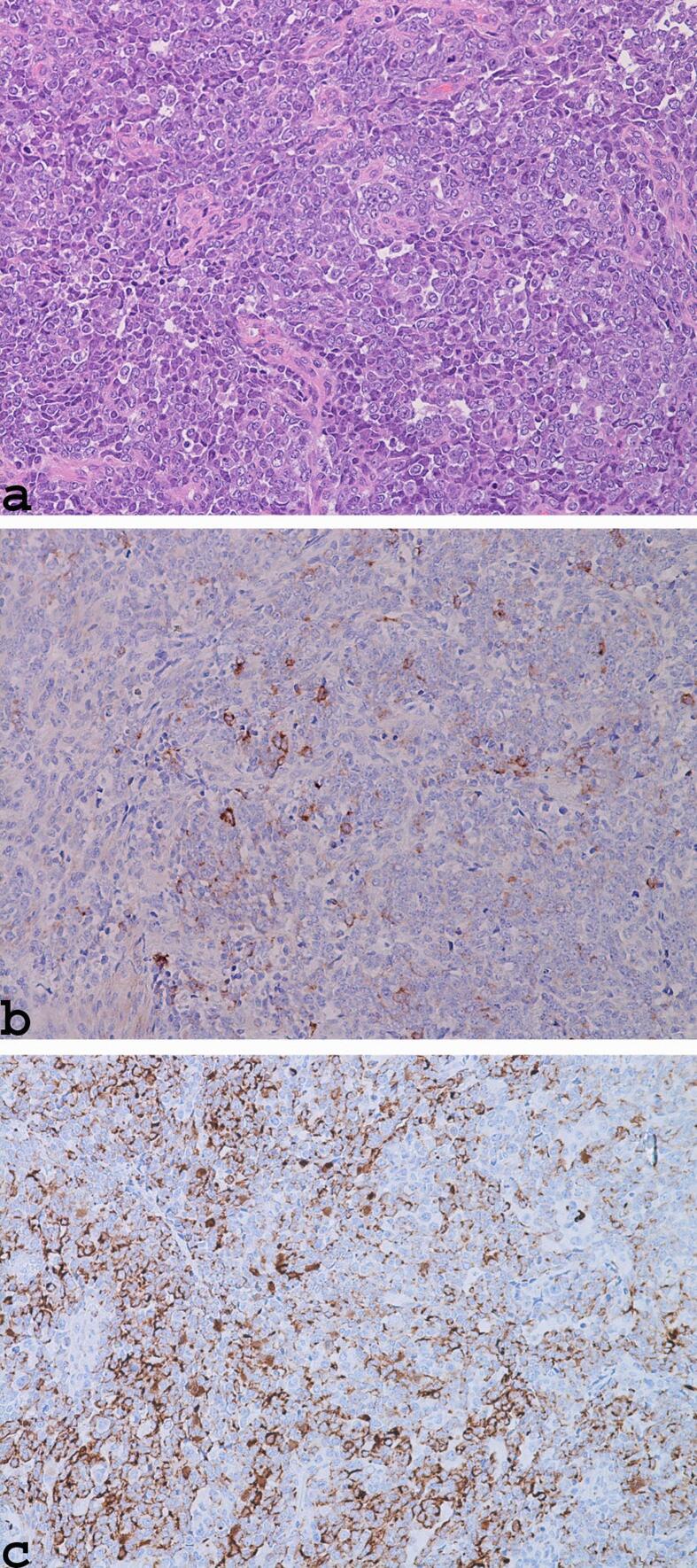
Histologically, endometrial neoplasm showing solid growth patterns with atypical, monomorphic, poorly cohesive neoplastic elements; nucleus with dispersed chromatin; evident basophilic nucleoli (a: haematoxylin and eosin stain, ×200); focal positivity for EMA (b: ×200); and focal positivity for CAM5.2 (c: ×200).

Histologically, endometrial neoplasm consists of solid growth patterns extending to the uterine isthmus; the neoplastic elements of this lesion are atypical, monomorphic, and sometimes poorly cohesive and showed a nucleus with dispersed chromatin, evident basophilic nucleoli, and high numbers of mitotic activities and apoptotic bodies (Fig. 2a). Immunohistochemical analysis showed that neoplastic cells detected focal positivity for epithelial membrane antigen (EMA) (Fig. 2b); focal positivity for CAM5.2 (Fig. 2c); focal positivity for PAX8; and negativity for LCA, ER, Pgr Ica, Ca 125, and neuroendocrine markers (synaptophysin and chromogranin A). These findings are consistent with the diagnosis of undifferentiated endometrial carcinoma.

**Fig. 3:**
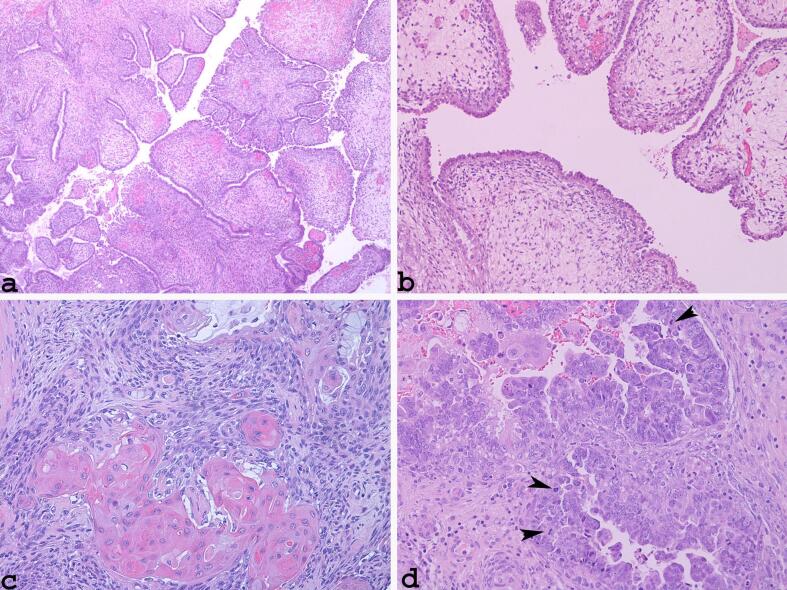
Polypoid lesion protruding from the cervical orifice (a: ×40) with oedematous stromal component below the lining epithelium from which it is separated by an evident and thin eosinophilic rhyme (band of respect) (b: haematoxylin and eosin stain, ×100). The epithelial component had squamous (c: haematoxylin and eosin stain, ×200) and glandular Müllerian mucinous differentiation (d: haematoxylin and eosin stain, ×200; arrow heads: mitoses).

The polypoid lesion protruding from the cervical orifice (Fig. 3a) had a biphasic appearance with a glandular Müllerian-type mucinous component and a stromal component; was partly oedematous (myxoid) with thin, branched vessels; and was partly dense with a concentric distribution around the glandular structures and below the lining epithelium, from which it was separated by an evident and thin eosinophilic rhyme (band of respect) (Fig. 3b).

The epithelial component had squamous and glandular Müllerian mucinous differentiation and showed (Fig. 3c, d) slight atypia and numerous mitotic activities. The oedematous stromal component is characterised by atypical fused or sometimes triangular cells. The neoplastic cells of the stromal component, which surrounded the glandular structures and were present under the lining epithelium, had a fused-oval shape. Their nuclei were atypical with moderate pleomorphism, dispersed chromatin, evident basophilic nucleoli, and frequent mitoses that were sometimes atypical. Immunohistochemical analysis showed that the glandular component had positive immunoreactivity for EMA (Suppl. 1a), CEA, and PAX-8 but had negativity immunoreactivity for CD10 and calretinin. The mesenchymal component showed diffuse positivity for vimentin, focal reactivity for CD 10 (Suppl. 1b) and smooth muscle actin (Suppl. 1c), and a high Ki67 index (Suppl. 1d) (>25% intra-nuclear positivity expression).

The part of the lesion that infiltrated and replaced the wall of the cervix with changes due to chemotherapy treatment, including marked hyaline fibrosis and inflammatory reaction with numerous foamy histiocytes, trapped the residues of small mucinous glandular structures.

Immunohistochemical examination indicated that the cervical neoplasm did not contain the endometrial epithelial component (undifferentiated carcinoma). Therefore, the post-operative histopathologic diagnosis was Müllerian-type cervical homologous CS that coexisted with undifferentiated endometrial carcinoma.

The cervical tumour had invaded almost the whole layer of the cervix but remained confined to the cervix. Neither lymphatic invasion nor vascular invasion of the tumour cells were noted. The pelvic and *para*-aortic lymph nodes and omentum did not show cervical CS metastasis. Conversely, the endometrial undifferentiated carcinoma had superficially invaded the myometrium and was associated with lympho-vascular space invasion (LVSI) and metastases both in the pelvic and *para*-aortic lymph nodes. Moreover, microscopic omental metastases of UCAe were found. Therefore, the final post-operative staging was (pT2a2, N0) for cervical malignancy and Stage IVB and T3a, N2, M1, LVSI, for UCAe respectively according to the FIGO system and TNM staging.

Following radical hysterectomy with lymph-node dissection, because of the advanced stage of development of neoplasm and for the presence of two highly aggressive components, the patient received adjuvant chemotherapy with carboplatin and Taxol for four cycles and pelvic and vaginal radiotherapy to avoid recurrence of the disease.

At the end of this treatment, CT study did not show macroscopic signs of disease recurrence.

However, the patient died after two months from post-actinic enteritis associated with intestinal occlusion.

## 3 Discussion

According to Warren and Gate (1932), the following criteria must be satisfied to define synchronous neoplasms in an organ:

• Each of the tumours must present a definite picture of malignancy.
• Each tumour must be clinico-pathologically distinct.
• The probability of each tumour being metastatic or recurrent must be excluded.

Synchronous primary tumours of the female genital tract are relatively rare and comprise only 1%–6% of all genital neoplasm cases. Endometrial cancer with synchronous ovarian cancer is the most common type of synchronous female genital tract malignancy and accounts for 50%–70% of cases (Ayhan et al., 1992, Tong et al., 2008, Eisner et al., 1989).

Uterine CS is a rare neoplasm, and it is even more rarely diagnosed as synchronous with other malignancies. Rare examples of primary squamous cervical carcinoma associated with adenocarcinoma and MMMT of the endometrium have been reported in literature in English (Ayhan et al., 1992, Eisner et al., 1989).

CS has been reported in the cervix (Kim et al., 2015, Iida et al., 2005). When this rare neoplasm is located in the cervix, it should be classified according to their origin as malignant mixed mesonephric tumours or MMMTs (Kim et al., 2015). Clinically, CS of the cervix is characterised by abnormal uterine haemorrhage, pelvic pain, dyspareunia, a polypoid or papillary cervical mass, or a large mass replacing the cervix.

In more advanced stages, patients present symptoms associated with the obstructive effect of the tumour on the gastrointestinal or urinary tracts (Piura et al., 2007).

The occurrence of primary cervical CS combined with another primary uterine malignancy is even rarer. In fact, upon reviewing literature in Medline via PubMed and Scopus by using the terms primary cervical CS, neoplasms, or malignancies associated with primary CS of the cervix, we found a few cases of cervical CS coexisting with other malignant primary cervical epithelial malignancies (Lin et al., 2017) and only one case in which homologous-type cervical CS coexisted with endometrioid-type endometrial cancer (G1) (Semczuk et al., 2014). To the best of our knowledge, the present case represents the first case in which two highly aggressive components were observed in the same uterus. The diagnosis of these components was formulated only on the histological examination of the hysterectomy specimen.

During the clinical examination, only the neoplasm protruding from the cervical orifice was evident.

Although MRI revealed the presence of endometrial mucosa thickening, it was not possible to evaluate this condition histologically because of the obstruction of the cervical canal due to the presence of simultaneous cervical neoplasia.

This case demonstrates that to indentify two distinct components in a neoplasm it is strongly encouraged an accurate immunohistochemical analysis with specific markers.

## Consent for publication

The parents of the patient agreed to the publication of these cases.

## Availability of data and materials

All data generated or analysed during this study are included in this published article.

## Funding

Not applicable.

## Authors contributions

The authors declare that they participated in this study as described below and have seen and approved the final manuscript. Giovanna Giordano was responsible for the concept of the paper, reviewed the available literature, evaluated the pathological data and drafted and edited the manuscript. Elena Feiroli reviewed the literature. Anna Maria Rodolfi evaluated the pathological data, Serena Madaro provided clinical data. Roberto Berretta performed the surgery and provided clinical data.

## Declaration of Competing Interest

The authors declare that they have no known competing financial interests or personal relationships that could have appeared to influence the work reported in this paper.

## Figures and Tables

**Supplementary Fig. 1:**
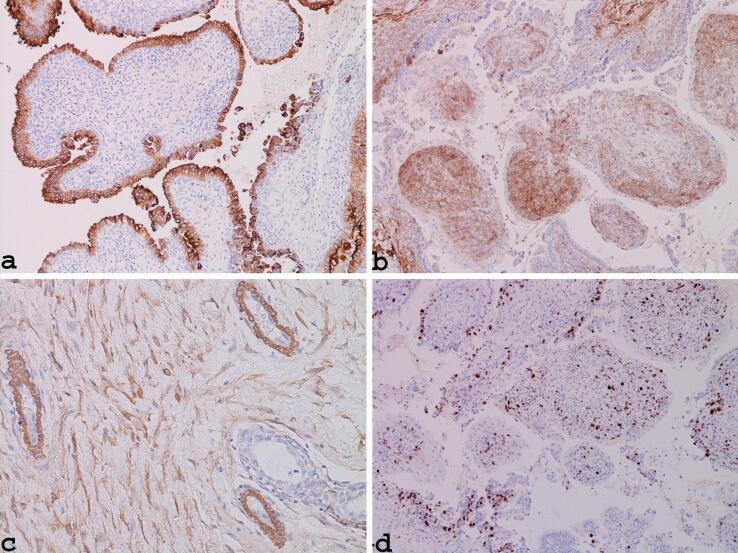
Immunohistochemical features of cervical neoplasm with diffuse positivity of the epithelial component for EMA (a: ×100), focal positivity for CD10 (b: ×100) and actin (c: ×200), and Ki67 index (d: ×200) of the stromal component.

## References

[b0005] Ayhan A., Yalcin O.T., Tuncer Z.S., Gurgan T. (1992). Synchronous primary malignancies of the female genital tract. Eur. J. Obstet. Gynecol. Reprod. Biol..

[b0010] Comert K.G., Turkmen O., Karalok A. (2017). Therapy modalities, prognostic factors, and outcome of the primary cervical carcinosarcoma: meta-analysis of extremely rare tumor of cervix. Int. J. Gynecol. Cancer..

[b0015] Eisner R.F., Nieberg R.K., Berek J.S. (1989). Synchronous primary neoplasms of the female reproductive tract. Gynecol. Oncol..

[b0020] Iida T., Yasuda M., Kajiwara H., Minematsu T., Osamura R.Y., Itoh J., Inomo A., Hirasawa T., Muramatsu T., Murakami M. (2005). Case of uterine cervical carcinosarcoma. J. Obstet. Gynaecol. Res..

[b0025] Kim M., Lee C., Choi H., Ko J.-K., Kang G., Chun K.-C. (2015). Carcinosarcoma of the uterine cervix arising from Müllerian ducts. Obstet. Gynecol. Sci..

[b0030] Lin Y., Chen H., Ye Z. (2017). Synchronous carcinosarcoma of the uterine cervix with adenoid basal carcinoma and cervical intraepithelial neoplasia III: A case report and literature review. Pathol. Res. Pract..

[b0035] Piura B., Meirovitz M., Shaco-Levy R. (2007). Carcinosarcoma of the uterine cervix initially interpreted as myoma nascens. J. Obstet. Gynaecol..

[b0040] Semczuk A., Colas E., Walczyna B. (2014). Coexistence of homologous-type cervical carcinosarcoma with endometrioid-type G1 endometrial cancer: a case report with an immunohistochemical study. Int. J. Clin. Exp. Pathol..

[b0045] Silva E.G., Deavers M.T., Bodurka D.C., Malpica A. (2006). Association of low-grade endometrioid carcinoma of the uterus and ovary with undifferentiated carcinoma: a new type of dedifferentiated carcinoma?. Int. J. Gynecol. Pathol..

[b0050] Silva E.G., Deavers M.T., Malpica A. (2007). Undifferentiated carcinoma of the endometrium: a review. Pathology..

[b0055] Tavassoli F.A., Devilee P. (2003). World Health Organisation Classification of Tumours. Pathology and Genetics. Tumours of the Breast and Female Genital Organs.

[b0060] Tong S.Y., Lee Y.S., Park J.S. (2008). Clinical analysis of synchronous primary neoplasms of the female reproductive tract. Eur. J. Obstet. Gynecol. Reprod. Biol..

[b0065] Warren S., Gate O. (1932). Multiple primary malignant tumors; survey of literature and statistical study. Am J Cancer..

